# Classification of Pathological Types of Lung Cancer from CT Images by Deep Residual Neural Networks with Transfer Learning Strategy

**DOI:** 10.1515/med-2020-0028

**Published:** 2020-03-08

**Authors:** Shudong Wang, Liyuan Dong, Xun Wang, Xingguang Wang

**Affiliations:** 1 Department of Respiratory Medicine, Shandong Provincial Hospital Affiliated to Shandong University, Jinan 250021, Shandong, China; 2 College of Computer and Communication Engineering, China University of Petroleum, Qingdao 266580, Shandong, China; 3 School of Electrical Engineering and Automation, Tiangong University, Tianjin 300387, China

**Keywords:** Pathological type, Lung cancer, Residual neural network, Transfer learning, CT images

## Abstract

Lung cancer is one of the most harmful malignant tumors to human health. The accurate judgment of the pathological type of lung cancer is vital for treatment. Traditionally, the pathological type of lung cancer requires a histopathological examination to determine, which is invasive and time consuming. In this work, a novel residual neural network is proposed to identify the pathological type of lung cancer via CT images. Due to the low amount of CT images in practice, we explored a medical-to-medical transfer learning strategy. Specifically, a residual neural network is pre-trained on public medical images dataset luna16, and then fine-tuned on our intellectual property lung cancer dataset collected in Shandong Provincial Hospital. Data experiments show that our method achieves 85.71% accuracy in identifying pathological types of lung cancer from CT images and outperforming other models trained with 2054 labels. Our method performs better than AlexNet, VGG16 and DenseNet, which provides an efficient, non-invasive detection tool for pathological diagnosis.

## 1 Introduction

Lung cancer accounts for more than a quarter of all cancer deaths. It is one of the major threats to human health on a worldwide scale [1]. In pathology, lung cancer can be mainly divided into two groups: small cell lung cancer (SCLC) and non-small cell lung cancer (NSCLC) [2]. NSCLC includes squamous cell cancer (SCC), large cell cancer and lung adenocarcinoma. Lung adenocarcinoma has two different types of adenocarcinoma in situ (ISA) and invasive adenocarcinoma (IA). The characteristics and treatments of different pathology subtypes of lung cancer are different.

Correct and timely diagnosis can implement an effective treatment plan and prolong patient survival. Nowadays, histopathology and molecular biology are standard for tumor pathological diagnosis, but usually can only be performed on excised tissue specimens such as surgical resection or needle biopsy [3]. However, radiology is a data-centric field involving the extraction and quantitative features to quantify the solid tumor radiographic phenotype [4]. It hypothesizes in [5] that radiographic phenotypes represent underlying pathophysiology, which shows different features in CT images.

Hardware advances in high resolution image acquisition equipment, coupled with novel artificial intelligence (AI) algorithms and large amounts of data, have contributed to a proliferation of AI applications in medical images. Convolutional neural network (CNN) has allowed for significant gains in the ability to classify images and detect objects from images [6, 7, 8, 9, 10, 11]. In lieu of the often-subjective visual assessment of images by trained clinicians, the deep learning method can automatically identify complex patterns [12, 13, 14, 15]. In [16], a sparse autoencoder with a denoising strategy (SDAE) was used to differentiate breast ultrasound lesions and lung CT nodule. A hierarchical learning framework with a multi-scale CNN was proposed to capture various sizes of lung nodules in [17, 18]. After that, a CAD framework with CNN is developed to classify the breast cancer in [19]. With the disclosure of lung nodule data sets and various challenges, lung nodule detection, segmentation, and classification algorithms emerged in an endless stream [20, 21, 22, 23].

A transfer learning strategy was proposed to deal with the limitation of data quantity, which is motivated by the fact that people can intelligently apply knowledge learned previously to solve new problems faster or with better solutions. We observe many examples of transfer learning in practice. Transfer learning was used in lung medical images processing to improve the classification accuracy of lung node assisted detection in [24]. Few transfer learning strategies focuses on the classification of lung cancer pathological types. To our best knowledge, there is no medical-to-medical transfer learning strategy, only learned representations usually being transferred from general imagery.

In this work, we propose a deep learning method with transfer learning strategy to identify pathology in types of lung cancer. Specifically, a novel residual neural network is proposed, and then a medical-to-medical transfer learning strategy is developed to process medical images, thus providing an accurate and timely diagnosis to the pathology type. Our residual neural network is pre-trained on public medical images dataset luna16, and then fine-tuned on our intellectual property lung cancer dataset collected in Shandong Provincial Hospital. Data experiments show that our method achieves 85.71% accuracy in identifying pathological types of lung cancer from CT images and outperforming other models trained with 2054 labels. Our method performs better than AlexNet, VGG16 and DenseNet, which provides an efficient, non-invasive detection tool for pathological diagnosis.

To our best knowledge, this is the first attempt at developing a medical-to-medical transfer learning strategy in classifying pathological types of lung cancer from CT images.

## 2 Methods

### 2.1 Materials

We use here two independent CT image datasets of lung cancer. The public dataset Luna16 is used for our model pre-training, i.e., the transfer learning. The other dataset of CT images of lung cancers on our intellectual property is collected from Shandong Provincial Hospital, and used for fine-tuning.

#### 2.1.1 Luna16

Luna16 is derived from the LUNA2016 dataset, which was originally created from the publicly available LIDC/IDRI database of lung CT scans. The LIDC/IDRI database contains nodule annotations which are collected during a two-phase annotation process using 4 experienced radiologists. LUNA2016 has organized much of the LIDC/IDRI database so as to make it readily available to groups working on medical imaging classifiers. LUNA2016 contains 888 CT scans which include 1186 nodules and provides 551,065 candidates to be classified. Each candidate has an , and position in coordinates and a classification as either non-nodular or nodular . It is noted that there can be multiple candidates per nodule. The dimensions of these images are 512512Z where Z is the varying in length depending on the height of patients scanned. We cut each candidate image to get the ROI region image of 5050 on the basis of the coordinates of the candidates.

#### 2.1.2 Our intellectual property lung cancer dataset

**Figure 1:**
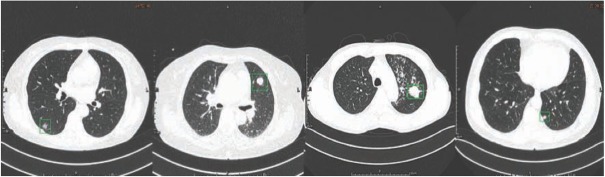
CT images of lung cancer pathological types: from left to right are ISA (adenocarcinoma in situ), SCLC (small cell lung cancer), SCC (squamous cell cancer) and IA (invasive adenocarcinoma). The green box areas are ROI areas of tumors.

**Figure 2:**
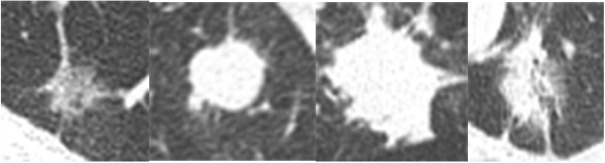
ROI areas of four types tumors, from left to right are ISA (adenocarcinoma in situ), SCLC (small cell lung cancer), SCC (squamous cell cancer) and IA (invasive adenocarcinoma).

We collected 125 chest CT scans cases with labeled tumors annotated by experienced Respiratory specialists from the Department of Radiology and the corresponding pathology reports in Shandong Provincial Hospital in 2018. It has obtained about 1500 CT images per lung cancer patient. Before training, each image goes through a tiered grading system consisting of multiple layers of trained graders of increasing expertise for verification and correction of image labels. The first-level scorer removes all low-quality or unreadable scans to screen all CT images for initial quality control, labeling tumor areas and manually cutting tumor areas to obtain ROI area images of 5050. The second tier of graders is composed by three Respiratory specialists who independently graded each image that had passed the first tier. They verified the true labels for each image. Finally, the data were classified into four types: ISA, SCLC, SCC and IA, which are shown in Figures 1 and 2.

### 2.2 Residual architecture

**Figure 3:**
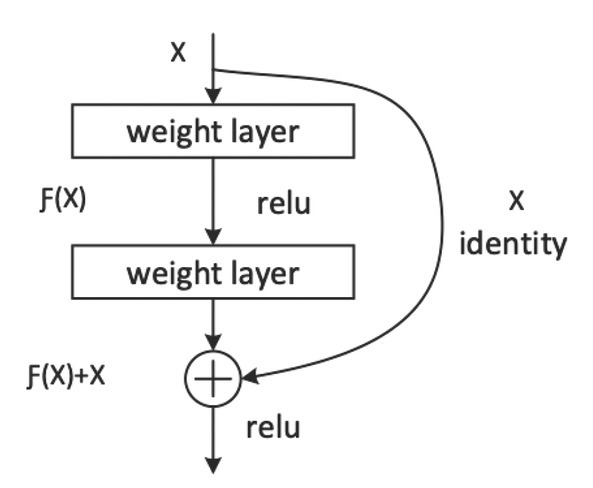
Schematic diagram of a residual block

In residual neural network, the residual learning is applied to every few stacked layers, which can relieve gradient disappearance and gradient explosion. A residual model is shown in Figure 3, where is the input value, and known as the residual, is the output after the first layer of linear change and activation. It is shown in Figure 3 that in the residual network, before the second layer is activated, adds the input value, and then the input of active function becomes. Adding X before the second activation is called a short cut connection. Residual networks allow training of deep networks by constructing the network through modules called residual models. Residual connections significantly boost the performance of deep neural networks.

**Figure 4:**
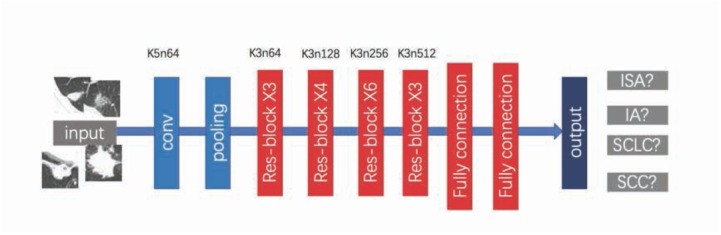
Architecture of our model which is based on residual blocks with corresponding kernel size, number of feature maps for each convolutional layer.

For lung cancer pathological types identification, we propose a novel depth residual network model, whose topological structure is shown in Figure 4.

Our model has a convolution layer with filters,16 residual units with filters, 2 fully connection layers along with their associated padding, pooling and dropout layers, and the structure of each residual unit is consistent with the structure shown in Figure 4. ReLU nonlinear activation function is used in all convolution, and then two fully connected layers is adopted to extract global features.

Our model differs from the traditional resnet-34 in the following three aspects:

1. We use kernels of size 5×5 for the first convolu-tional layer, while the original resnet-34 uses kernels of 7×7 for the first convolutional layer. This is due to the size of images in our database (50×50), so we chose kernels of 5×5. And in the experiment, we found that kernels of 5×5 perform better than kernels of 7×7.
2. Our model has two full connection layers. The original resnet-34 has only one full connection layer. Since we use the transfer learning strategy, it needs to design two full connection layers for improving the migration ability of the model. Full connection layers are seen as a “firewall” for model representation capabilities. For fine-tuning, the result of network fine-tuning without a full connection layer is worse than that of a network with a full connection layer. In order to improve the ability of model migration, we designed two full connection layers here.
3. In our model, the SIGMOD activation function is used in pre-training, and SoftMax activation function is used in fine-training in the full connection layer.

The final output layer predicts the type of input image, as an input to the class loss term and the centering loss term during training. In dense layers, we have two options for activation function:

1. SoftMax activation function with categorical cross-entropy loss function and one-hot encoded inputs
2. Sigmoid activation function with binary cross-entropy loss function.

In pre-training, we select the sigmoid activation function with binary cross-entropy as loss function, thus outputting an integer value of 0 or 1 depending on the predicted output. In fine-tune, we use the SoftMax activation function with categorical cross-entropy loss function and one-hot encoded inputs, to output the probabilities of pathological types. The retrained model with the least validation loss is obtained on our own dataset.

### 2.3 The transfer learning strategy

Our model is trained with the transfer learning strategy on a public dataset. In detail, our model is pre-trained on the luna16 dataset by using keras. In pre-training, the model parameters are randomly initialized, then the whole model is trained and the weights are saved. It has achieved accuracy rate 96.69% on luna16. In fine-tuning, the model parameters are initialized to the weights saved after pre-training. Specially, the first 27 convolutional layers of model are frozen with loaded pre-trained weights, the remaining convolutional layers and fully connection layers are retrained to recognize our classes from scratch. In identifying lung cancer pathological types from Our intellectual property lung cancer dataset, the first 27 convolutional layers are frozen and used as fixed feature extractors. The previous 27 layers are used to extract generic features (such as edge detection and color detection) of images. The latter layers are utilized to abstract features related to a particular category.

**Figure 5:**
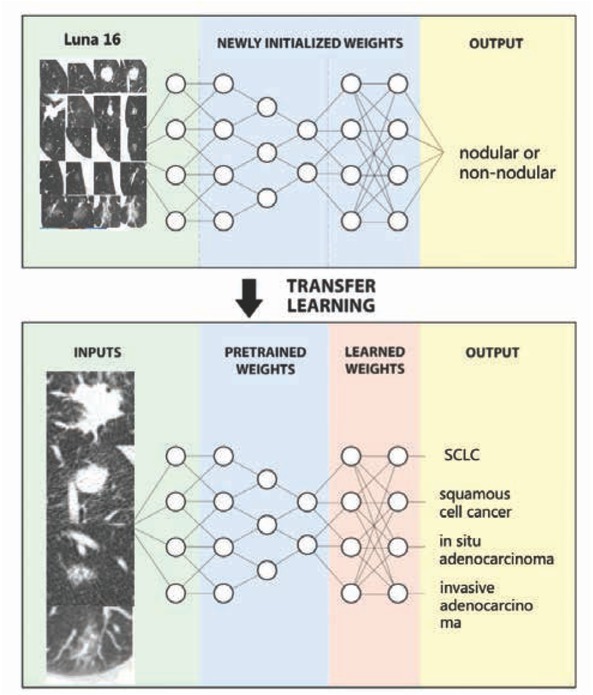
The general framework of the transfer learning strategy. The upper part is pre-training, and the lower part is fine-tuning. When we do fine-tune process, we update the weights of some layers.

Our transfer learning strategy attempted to fine-tune the last 8 layers. The convolutional “bottlenecks” are the values of each training and testing images after they have passed through the frozen layers of our model. Since the convolutional weights cannot be updated, such values are initially calculated and stored in order to reduce redundant processes and speed up training. The newly initialized network takes the image bottlenecks as input and retrains to classify our specific categories, see Figure 5. Attempts at ‘fine-tuning’ model by unfreezing and updating the pre-trained weights using backpropagation tend to decrease model performance due to overfitting on our medical images.

### 2.4 Model details

Since the size of the Luna16 dataset is substantial, it is impossible to load the whole set into memory. We opted for batch processing of the “.png” files using Keras’s ImageGenerator function while generating from directory. There are two-fold advantages: image size is verified and resized before feeding into the network and no manual loading is required to feed pixels into the neural network. The main drawback is the complexity of cross-validation because it did not support automatic partitioning of the data. Partitioning for validation is done manually here.

The lung cancer dataset is unbalanced in each type of data. After data preprocessing, 67 SCLC images, 98 SCC images, 1818 IA images and 71 ISA images are obtained for training. Since the number of IA images is about 20 times that of other categories, we divided the 1818 IA images into 20 groups, each of which has about 90 images to alleviate the imbalance. In each epoch, only one of the 20 sets is randomly chosen as a part of training set.

Our model is pre-trained on Ubuntu 16.04 OS with 2 Intel Xeon CPUs, using a NVIDIA GTX 2080 8Gb GPU for training and testing, 256Gb RAM, NVIDIA GTX 1080 4Gb GPU for fine-tune.

Pre-training of layers is performed by a stochastic gradient descent in batches of 32 images per step using an Adam Optimizer with a learning rate of 0.001. Since the pre-trained weights are better than the random initialization weights, we set a smaller learning rate. Fine-tuning of layers is performed by stochastic gradient descent in batches of 20 images per step using an Adam Optimizer with a learning rate of 0.0001. Pre-training and fine-tune on all categories are both run for 100 epochs, since training of the final layers can converge for all classes. Holdout method testing is performed after every step by delivering images to network without performing gradient descent and back propagation. The model with the best performance is kept for analysis.

Informed consent: Informed consent has been obtained from all individuals included in this study.

Ethical approval: The research related to human use has been complied with all the relevant national regulations, institutional policies and in accordance the tenets of the Helsinki Declaration, and has been approved by the authors' institutional review board or equivalent committee.

## 3 Results

The dataset of images for training and testing images is tabulated in Table 2. Specifically, 2054 images are used for training and the remaining 168 images are used for testing. The training set includes 67 SCLC images, 98 SCC images, 1818 IA images and 71 ISA images. The test set holds 29 SCLC images, 43 SCC images, 65 IA images and 31 ISA images.

**Table 1:** Parameters during pre-training and fine-tuning

	Pre-train	Fine-tune
Hardware	NVIDIA GTX 2080 8Gb GPU	NVIDIA GTX 1080 4Gb GPU
Batch size	32	20
Epoch	100	100
Learning rate	0.001	0.0001

**Table 2:** The data-based image for training and testing

Class	Training	Testing
SCLC	67	29
SCC	98	43
IA	1818	65
ISA	71	31
Total Images	2054	168

**Figure 6:**
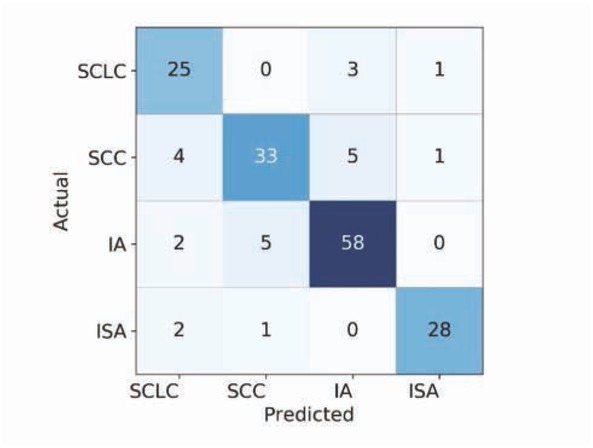
Confusion matrix of test result.

The average accuracy is 85.71% after 5 runs. The test results of one run of our method are illustrated by a confusion matrix, as shown in Figure 6.

For the SCLC images, 29 images are used for testing, among which 25 images are correctly predicted, 3 images are misclassified as ISA, and 1 image is misclassified as IA. For SCC images, 43 images are used for testing, among which 33 images can be correctly predicted, and 4 images are misclassified as SCLC, 5 images are misclassified as ISA and 1 image is misclassified as IA. For IA images, 65 images are used for testing, among which 58 images are correctly predicted as IA, 2 images are misclassified as SCLC and 5 images are misclassified as SCC. For ISA images, 31 images are tested, in which 28 images are correctly predicted as ISA, 2 images are misclassified as SCLC and 1 image is misclassified as SCC.

From the results, it was found that our model is capable of predicting the medical conditions of lung cancer. It is shown in Table 2 the accuracy level of the lung cancer image classification rate of the proposed method. Our transfer learning strategy can identify the four lung cancer pathological types from CT images. The results showed that the model has a slightly higher ability to distinguish IA from other categories, which may be due to the fact that IA type data is much more than other types of data.

**Table 3:** Proposed CT lung image classification with transfer learning results

Type	Recall	Precision
ISA	0.8923	0.8787
SCLC	0.8621	0.7575
SCC	0.7674	0.8461
IA	0.9032	0.9333

**Figure 7:**
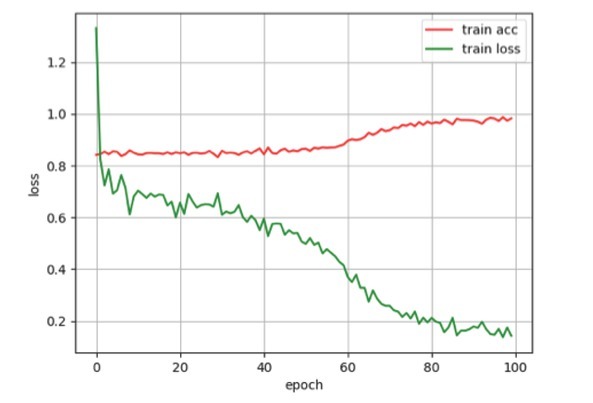
Training accuracy and cross-entropy loss are plotted against the training epoch. Plots were normalized with a smoothing factor of 0.5 to clearly visualize trends.

In Figure 7, it provides an analysis of the accuracy and loss values for the network architecture during the training period with the transfer learning. With the benefit of transfer learning, our model achieved high precision at early epochs.

Binary classifiers are also implemented to compare SCLC/ squamous cell cancer/ invasive lung adenocarcinoma from each other. The same datasets are used to determine a breakdown of the model’s performance. The classifier distinguishes SCLC images from squamous cell carcinoma images achieved an accuracy of 94.5%, with a sensitivity of 100.0% and specificity of 89.65%. It distinguishes SCLC images from invasive lung adenocarcinoma images achieved an accuracy of 93.25%, with a sensitivity of 93.1% and specificity of 93.84%. It achieved an accuracy of 95.75%, with a sensitivity of 95.34% and specificity of 96.92% to distinguish squamous cell carcinoma images from invasive lung adenocarcinoma images.

We compare our approach with some classical statistical machine-learning classifiers and recent state-of-the-art methods to prove the superiority of our method. The AlexNet used in this experiment includes 5 convolution layers and 3 fully connection layers. The DenseNet has 121 layers with 4 dense blocks. The original VGG16 Network includes 13 convolution layers with filters and 3 full connection layers. We find that 5 runs is sufficient to prove the performance of the model. All the comparative models were trained using our lung cancer data without transfer learning. The dataset of images for training and testing images is tabulated in Table 2. It is shown in Figure 4 that AlexNet trained on our own dataset resulted in the relatively low accuracy of 63.98%, DenseNet resulted in the accuracy of 80.7%, VGG16 resulted in accuracy of 78.42%. Our model without transfer learning gets 79.53% accuracy.

**Table 4:** Results of binary classifiers based our model

Binary classifiers	accuracy	sensitivity	specificity
SCLC/SCC	0.9450	1．00	0.8965
SCLC/IA	0.9325	0.9310	0.9384
SCC/IA	0.9575	0.9534	0.9692

**Table 5:** Accuracy (%) on our dataset over 5 runs

AlexNet[26]	VGG16[27]	DenseNet[28]	Ours without transfer learning	Ours with transfer learning
63.980.10	78.42	80.7.89	79.532.64	85.71.29

These results indicate that the misdiagnosis rate was high and that a number of lung cancer cases were not discriminated. In conditions with a limited training set, classical machine-learning models cannot be effectively trained. These results show that our method resulted in better performance than the others.

## 4 Discussion

This study used CT images to analyze and identify the pathological types of lung cancer, which is non-invasive, specific and reproducible. We describe a general AI model for the diagnosis of pathological subtypes of lung cancer. By employing a transfer learning strategy, our model demonstrated competitive performance of CT image analysis without the need for a highly specialized deep learning machine and without a database of millions of example images. Moreover, the model’s performance in diagnosing lung cancer CT images was comparable to that of human experts with significant clinical experience with lung cancer, outperforming other methods. When the model was trained with a much smaller number of images(about 100 images of each class), it retained high performance in accuracy, sensitivity, specificity for achieving the correct diagnosis , thereby illustrating the power of the transfer learning system to make highly effective classifications, even with a very limited training dataset.

Our AI model was trained and validated on our intellectual property lung cancer dataset collected in Shandong Provincial Hospital, but the Digital Imaging and Communications in medicine standards cause inconsistencies in CT images from different manufacturers. Future studies could entail the use of images from different manufacturers in both the training and testing datasets so that the system will be universally useful. Moreover, the efficacy of the transfer learning technique for image analysis very likely extends beyond the realm of CT images and lung cancer. In principle, the techniques we have described here could potentially be employed in a wide range of medical images across multiple disciplines.
